# Patient-Reported Barriers to Foregut Cancer Care in the Deep South

**DOI:** 10.1245/s10434-025-17113-2

**Published:** 2025-03-04

**Authors:** Ioannis Liapis, Alfonsus Adrian H. Harsono, Jaspinder Sanghera, Katie West, Rida Ahmad, Michelle Holland, Larry Hearld, Smita Bhatia, Krista Mehari, Martin J. Heslin, Daniel I. Chu, Annabelle L. Fonseca

**Affiliations:** 1 Department of Surgery, University of Alabama at Birmingham, Birmingham, AL USA; 2 Department of Psychology, University of South Alabama, Mobile, AL USA; 3 Department of Surgery, University of South Alabama, Mobile, AL USA; 4 Department of Health Services Administration, University of Alabama at Birmingham, Birmingham, AL USA; 5 Institute of Cancer Outcomes and Survivorship, University of Alabama at Birmingham, Birmingham, AL USA; 6 Department of Psychology, Vanderbilt University, Nashville, TN USA

**Keywords:** Foregut cancer, Guideline concordant treatment, Social determinants of health, Mixed methods, South

## Abstract

**Background:**

Many patients with foregut cancer do not receive guideline-concordant treatment (GCT). Although social determinants of health (SDOH) have been associated with differences in receipt of GCT, the underlying mechanisms that perpetuate these disparities remain unknown. This mixed-methods study explored barriers to receipt of care among patients with foregut cancer.

**Methods:**

Patients with foregut cancers treated at a safety-net hospital in the Deep South were purposively selected. The patients completed semi-structured interviews, which were recorded, transcribed, and analyzed. Grounded theory methodology was used to generate themes through open coding, develop a thematic coding structure, and create a codebook. Intercoder agreement was above 90%. Patient sociodemographic and treatment-related variables were abstracted from the patients’ medical records to produce simple descriptive statistics.

**Results:**

The majority of the 30 participating patients were male (*n* = 23, 77%), black (*n* = 18, 60%), and with a median age of 63 years (interquartile range, 55–67 years). Using the socioecologic model, barriers were categorized into individual, interpersonal, organizational, and policy levels. Within the individual level, the barriers were access to primary care providers, personal barriers, competing responsibilities, multifaceted financial barriers, and transportation barriers. The interpersonal barriers involved communication challenges, physician mistrust, and absence of social support. The organizational level barriers were health system mistrust, inadequate health care infrastructure, and lack of insurance coverage consequences. The policy level barriers were health care access policies and insurance policies.

**Conclusions:**

The patients reported multiple barriers related to accessing and adhering to their treatments. Understanding these barriers is critical to forming the basis for developing and implementing programs to increase the delivery of GCT.

**Supplementary Information:**

The online version contains supplementary material available at 10.1245/s10434-025-17113-2.

Foregut cancers (i.e., cancers of the esophagus, stomach, pancreas, liver, and biliary system) are associated with poor overall survival due to both underlying tumor biology and challenges to the delivery of cancer care.^1–5^ Guideline-concordant therapy (GCT) (i.e., tumor type and stage-specific standard-of-care treatment) has been demonstrated to improve outcomes in foregut cancer. However, studies report that up to 70% of patients do not receive GCT. Non-receipt of GCT is associated with decreased overall survival.^6,7^ Although outcomes are partly influenced by underlying tumor biology, the impact of social determinants of health (SDOH) is unequivocally significant along the continuum of foregut cancer care.^8,9^ Race, socioeconomic status, and residence in specific geographic or rural locations, or in areas of high disadvantage, as measured by the Area Deprivation Index (ADI), are associated with decreased receipt of GCT.^10,11^

The treatment of foregut cancers is inherently challenging. Whereas systemic therapy is the mainstay of treatment, with most patients presenting with metastatic disease, non-metastatic disease is treated with multimodal therapy combining curative-intent surgical resection and chemotherapy. Guideline-concordant therapy requires coordinated and multidisciplinary care, part of which is delivered at tertiary care centers. Challenging chemotherapy regimens and technically complex operations, together with the cancer-related deconditioning that often occurs, pose additional challenges to the delivery of GCT.

Much of the existing literature involves quantitative studies showing the association of SDOHs with disparities in access to care and outcomes. However, a gap in the literature remains regarding the underlying causes for these associations. Qualitative and mixed-methods research plays an important role in understanding the underlying mechanisms contributing to cancer care disparities. The socioecologic model categorizes barriers to health outcomes across multiple levels (individual, interpersonal, organizational, and policy) and was used to capture the interplay of factors associated with health disparities.^12,13^

Gaining insight into the “why” from the patient perspective is key to a more comprehensive understanding of the barriers that exist and what interventions may be developed to target them. To this end, we sought to explore and understand patients’ perspectives on barriers to access and adherence to cancer care among patients with foregut cancer treated at a safety-net hospital in the Deep South.

## Methods

### Study Design and Setting

This study used a convergent mixed-methods design, in which qualitative data from patient interviews and quantitative data obtained through in-depth review of electronic health records were collected in parallel, analyzed separately, and then merged for interpretation.^14^ The study was conducted at a 650-bed tertiary referral center in the southeastern United States that serves as the safety-net hospital for central and southern Alabama, as well as for nearby areas including southeast Mississippi, parts of Louisiana, and northwest Florida. The overall catchment area encompasses a racially diverse population of approximately 5–6 million people,^15^ and the health system provides care to approximately 200–300 patients a year with foregut cancer.

The study protocols and all associated materials were approved by the University of South Alabama (USA) Institutional Review Board (IRB-2003085-1). The study adhered to the consolidated criteria for reporting qualitative research (COREQ)^16^ guidelines, and the checklist is provided in Supplementary 1.

### Participant Recruitment and Interview

Adult patients of any race or gender with gastric, esophageal, pancreatic, or hepatobiliary cancer at any stage who were English-speaking and received treatment at the cancer center between May 2022 and May 2023 were eligible for inclusion. Patients were purposively sampled based on a combination of factors including type and stage of cancer, geographic location, and self-reported barriers to care. This sampling strategy ensured diverse representation of patient experiences while allowing for a focused exploration of key barriers.

Patients were approached in clinic by a member of the research team not involved in clinical care. Of the 35 patients approached, 30 consented to participate, and 5 declined due to time constraints. The patients participated in in-person, semi-structured interviews, which lasted 30 to 60 min, conducted at a time convenient to the participants in a private location within the cancer center, with their caregiver present if they requested. All the study participants provided informed consent for the interviews and received financial compensation of $25 for their time.

The semi-structured interview guide was developed based on grounded theory methodology to capture voices of the patients and their experiences of barriers in access to cancer care across the health system. The interview guide was pilot-tested with cancer patients before formal data collection to assess clarity and ensure relevance of the questions. The interview guide is included as Supplementary 2. Open-ended questions and probing responses were included to encourage dialogue.

Two trained interviewers (K.W., R.A.) conducted all the interviews. The interviewers were two graduate female students who had no prior relationships with the participants and no known preconceived biases on the subject. The interviewers were trained in semi-structured interviewing by a clinical psychologist and an experienced member of the research team. Each interviewer completed two practice interviews that the investigators listened to, then provided written and oral feedback. Interview tapes and field notes made during the interview were reviewed throughout the study to mitigate interview drift and ensure accuracy. No repeat interviews were deemed necessary, and the patients did not receive a transcript of their interview nor provided feedback on the results of the study. Interviews were conducted until thematic saturation was achieved.

### Quantitative Data Collection; Patient and Procedure Variables

Patient sociodemographic and treatment-related variables were abstracted from electronic health records including age, race, sex, insurance status, presence of a primary care provider (PCP), time from onset of symptoms to initiation of diagnostic workup, time from diagnosis to first oncology appointment, and time from oncology appointment to treatment initiation. Records also were reviewed thoroughly to identify whether patients received GCT, as defined based on the National Comprehensive Cancer Network (NCCN) guidelines.

In all cases, GCT was determined by review of medical records to account for adherence to NCCN type- and stage-appropriate guidelines, with consensus regarding definition of GCT achieved by two surgical oncologists. The definition of GCT differed depending on the type of cancer and stage at presentation, as described in a previous study. Neighborhood disadvantage also was assessed based on the ADI, a validated neighborhood-level composite index based on 17 specific measures captured in the American Community Survey.^17^ As a composite index, ADI allows for ranking neighborhoods based on socioeconomic disadvantage. The state-level ADI is a decile ranking at the block group level from 1 to 10, with group 1 indicating the lowest level of disadvantage and group 10 indicating the highest level of disadvantage within the state. For the purpose of this study, state deciles were grouped into the following tertiles: low, intermediate, and high deprivation. Simple descriptive statistics were generated as appropriate.

### Qualitative Analysis

All the interviews were audio-recorded, transcribed verbatim, and reviewed for accuracy, and de-identified. Three coders (K.W., R.A., A.F.) independently analyzed and coded verified transcripts using NVivo 14 software (QSR International Burlington, MA, USA), a multifunctional platform widely used for qualitative data analysis. Qualitative data were analyzed using grounded theory methodology, which emphasizes the emergence of themes from participant experiences rather than a predetermined theoretical framework. This method was adopted instead of more organizationally focused implementation science frameworks to emphasize and center the patient experience.^18^

Open coding was first performed to generate preliminary themes. The coders used a consensus approach and grounded theory to finalize themes and develop a thematic coding structure in secondary coding. Themes were mapped to the four levels of the socioecologic model^15^ (individual, interpersonal, organizational, and policy) to facilitate interpretation within a structured, multi-level framework. The consensus around definitions and structure was used to create a codebook with definitions that guided tertiary coding.

Throughout the analysis, a constant comparative method was used to guide an inductive analytical process and ensure saturation in the data.^19^ This process allowed for the identification of common themes found among the interview participants while simultaneously capturing varied perspectives on discussed topics. Regular meetings and discussions among the coders were conducted to reach an interrater reliability above 90%.^20^

### Integration of Data

A convergent mixing approach was used to integrate qualitative themes with quantitative patient data. Data reduction and data display were performed during this phase. Triangulation of qualitative themes with patient-level variables was performed to identify patterns. Additionally, qualitative themes were quantitized by converting thematic data into categorical variables to explore how their distributions differed from the marginal distributions across levels of the quantitative variables. This integration allowed for a richer understanding of barriers to GCT by contextualizing patient-reported experiences within their clinical and sociodemographic realities.

## Results

The study enrolled 30 patients. The majority of the participants were male (*n* = 23, 77%), black (*n* = 18, 60%), and residents in areas of high deprivation (*n* = 19, 63%). The median age was 63 years (interquartile range [IQR], 55–67 years). Most of the patients were receiving treatment for pancreatic cancer (*n* = 13, 43%), followed by gastric cancer (*n* = 9, 30%) and liver cancer (*n* = 8, 27%). Overall, 18 (60%) of the patients interviewed had a PCP. The median time from symptom onset to diagnostic workup was 10 weeks (IQR, 6.1–15.6 weeks). The median times from diagnosis to oncology appointment was 6 weeks (IQR, 4.7–7.3 weeks) and from oncology appointment to treatment initiation was 5 weeks (IQR, 3.4–6.1 weeks). Descriptive statistics are presented in Table 1.

**Table 1:** Characteristics of participants

Characteristics	(*n* = 30) *n* (%)
*Sociodemographic variables*	
Median age: years (IQR)	63 (55–67)
*Race*	
Black	18 (60)
White	12 (40)
*Gender*	
Female	7 (23)
Male	23 (77)
*Insurance*	
Private	6 (20)
Medicare	11 (37)
Medicaid	3 (10)
VA	3 (10)
Uninsured	7 (23)
*Area deprivation*	
Low	4 (13)
Intermediate	7 (23)
High	19 (63)
*ECOG*	
0–1	21 (70)
≥ 2	9 (30)
*Cancer type*	
Pancreatic	13 (43)
Gastric	9 (30)
Liver	8 (27)
*Access-related variables*	
Receipt of guideline-concordant treatment	15 (50)
Presence of PCP	18 (60)
Median time from symptom onset to diagnostic workup: weeks (IQR)	9.7 (6.1–15.6)
Median time from diagnosis to oncology appointment: weeks (IQR)	5.9 (4.7–7.3)
Median time from oncology appointment to treatment: weeks (IQR)	5.0 (3.4–6.1)

IQR, interquartile range; VA, veterans affairs; ECOG, Eastern Cooperative Oncology Group; PCP, primary care physicians

### Category 1: Individual-Level Barriers

Within the individual level, five themes were identified. These themes with the percentage of patients reflecting them in descending order were transportation barriers (77%), multifaceted financial barriers (50%), competing responsibilities (43%), access to PCP (30%), and personal barriers (17%). Regarding the transportation barriers, individual patients described difficulties related to transportation distance, logistics, and cost. Several patients reported having to travel long distances to access specialized cancer care: “Just to drive down here all the time, staying overnight, having early morning appointments, I have to drive almost a hundred miles to get here” (Q14). Arranging transportation often required relying on family members, adding a logistical challenge: “It’s not easy. Sometimes my brother or sister-in-law or somebody will bring me.” (Q13). Patients also reported the cost of transportation as a barrier to access: “… it’s the gas prices and wear and tear on your vehicle. It's been tough.” (Q15).

Financial strain was reported by half of the participants. Patient and caregiver wage loss as well as the cost of treatment had an impact on patients’ ability to pay for treatment and meet basic needs. For example, one participant expressed the difficult trade-offs encountered: “You have to be thinking what is the next thing you need do, pay your (medical) bill or put gas in your car” (Q9). The participants also described financial strain due to their caregivers’ wage loss from needing to reduce work hours or stop work entirely: “… my wife can’t work because she’s bringing me back and forth to the doctors” (Q11). The high cost of treatment compounded these challenges, as another patient noted: “The only thing that harms me is the bill that I get in the mail” (Q12).

Competing responsibilities were another major theme, with participants reporting juggling caregiving duties for children, grandchildren, or elderly family members. Some participants reported that these obligations left them feeling overwhelmed and unsupported as they navigated their cancer journey. Two thirds of the patients who reported barriers related to competing responsibilities did not receive GCT. Access to PCPs was reported as a barrier by 30% of the participants. Notably, among the 40% of patients without a PCP, less than half reported it as an issue (Table 2).

**Table 2:** Socioecologic model of reported barriers to diagnosis and access to cancer care

Socioecologic-level/themes	Percentage of patients^a^	Sub-themes	Quotes
*Individual*			
Access to PCP	30%	Personal choice	Q1: “Now, if I am not sick, I don’t need to go to no doctor. When I’m sick, then I know I need to go.”
		Delay in diagnosis	Q2: Interviewer: “How long were you unwell before you found out that you had cancer?” Participant: “About 3 months.” Interviewer: “During these 3 months, were you in and out of the hospital or seeing doctors?” Participant: “No. I was trying to get into see doctors, because I didn’t have a primary care doctor.”
Personal barriers	17%	Reluctance or hesitation in seeking medical care	Q3: “I was real sick having stomach issues and a lot of pain in my stomach. I wouldn’t go to the doctor, and my girlfriend kept trying to get me to go to the doctor. I said, no, I know something’s really bad wrong, and I don’t want to. Men are stubborn about going to the doctor, and I kept refusing and refusing.”
		Cultural or personality factors	Q4: “We do get some help, but not much. That’s because we’re proud people. We don’t ask for that much help, but since this has happened, it’s been hard, and it’s been hard not to ask.”
		Substance use impacting health-seeking behavior	Q5: “That’s a pain I don't wish for anybody. Dealing with cocaine, alcohol, weed and all that stuff. That’s what putting us into your hospital; alcohol, major drugs and stuff like that. That’s why we are sick.”
Competing responsibilities	43%	Caregiving duties	Q6: “My mother she’s disabled, and I’m taking care of her down in the household, and we have to feed her, and we have to bathe her and everything.”
		Financial obligations	Q7: “I have to pay my bills at home to keep a roof over me and my kids’ heads. I don’t know. It’s just we need more help with that.”
		Sacrifices made by caregivers	Q8: “… my sister, she’s dealing with her husband because he has one leg, so she’s been awesome. Tremendous help, and she still tries to work in between taking him to the doctor. If it doesn’t work for her taking me or him to the doctor, we work it out. I have my son. He’ll come and take me, but he works, but he’ll take off or take me, do what he has to do…”
Multifaceted financial barriers	50%	Personal income loss	Q9: “And that’s (wage loss) a lot of problem, and they let you sit back and pull your hair out of your head and, thinking what is the next thing you need to do first, pay your bill or put gas in your car. I shouldn’t have that thought.”
			Q10: “I can’t work… I have not received a check since November, so we're back to square one of just trying to survive.”
		Caregiver’s income loss	Q11: “I can’t work since they diagnosed me, and my wife can’t work, because she’s bringing me back and forth to the doctors.”
		Costs of treatment	Q12: “The only thing that harms me is the bill that I get in the mail.”
Transportation barriers	77%	Transportation logistics	Q13: Interviewer: “How easy is it to get someone to have you drive down here?” Participant: “12031: It’s not easy. Sometimes my brother or sister-in-law or somebody will bring me.”
		Transportation distance	Q14: “Just to drive down here all the time, staying overnight, having early morning appointments, I have to drive almost a hundred miles to get here.”
		Transportation cost	Q15: “We’ve had to come over here twice a week to get either a procedure or anything, but mostly it’s the gas prices and wear and tear on your vehicle. It’s been tough.”
*Interpersonal*			
Communication challenges	47%	Use of complex medical terminology	Q16: “When he tells me what’s going on, I don’t… understand it in doctor’s terms.”
		Information overload	Q17: “More explanation when you quit seeing one doctor and get turned over to another doctor… You just lose track of your doctors in the process.”
		Inadequate physician communication	Q18: “After talking with Dr. (surgeon), I’m kind of disappointed that they didn’t tell me that I did have an option of surgery before now. In the earlier stages, seven months ago, six months ago or whenever.”
		Lack of clarity of information provided	Q19: “I saw them a year prior to it, and it was a spot; but then he saw it again, and he said it’s bigger. I said, something’s wrong, then. He said, ‘Just wait and see in with your primary care’. Then she called me to have an appointment with (Dr. name), who’s an oncologist. And I said, so I have cancer? She said, "Yes, you do.”
Mistrust toward physicians	20%	Past negative experiences	Q20: “He said everything was okay. He didn’t find anything, but I know his diagnosis or whosoever diagnosis was it, it wasn't right. There’s still something going on. So then, I came here, and they did what they did. Still came up the same thing… I wasn't satisfied still, so that’s … when he found what he found. I didn’t get satisfied until they found what they had to find and that was at urgent care. I wasn’t satisfied with the answers because I know, in my body, it was telling me something wasn’t right.”
		Perceived inconsistencies in care	Q21: “We were misinformed a long time ago about the surgery…. They said it wasn’t curable, then she said it was… you get two different stories from the whole situation…”
		Lack of connection with providers	Q22: Q: “How much trust do you have for your specific doctors?” A: “So, like which one? I don’t have doctors really truly, because I don’t even know their names. I go to the clinic out here. I see a lady doctor at the clinic, and sometimes it will be a man. They are not there long enough to really get to know them, because I don’t really get sick.”
Absence of social support	27%	Perceived isolation or lack of support systems	Q23: “I’ve got my phone, … but let’s face it, my phone doesn’t ring that much. I don’t have a lot of people that I talk to.”
*Organizational*			
Health system mistrust	37%	Negative past experiences	Q24: “The team that I got now has been great. It’s just before, nobody really cared.”
		Mistrust toward hospital	Q25: “[I’m] leaving the hospital with a prescription, I don’t believe I’ll get that prescription filled. I have no faith in the hospital. When I leave there, I’m on my own. I’m on my own most of the time I’m there too. So, no hospitals aren’t thrilling me.”
		Lack of symptoms management	Q26: “I was in a whole lot of pain, and even though they say the test says you’ve got cancer, they wouldn’t do anything for pain. They made me appointment a week away and wouldn’t do anything for that week. We just diagnose, we don’t give you any pain pills. So, it was liked what do I do?”
			Q27: “A guy at clinic told me one day, the only reason you come here is for the Medicaid, I said, no, I came here because I’m sick. I'm hurt. And do you know, he didn’t give me anything.”
Inadequate healthcare infrastructure	37%	Difficulty accessing primary care	Q28: I need one (primary care physician), but I don’t have one now. I don’t know the way to get one.”
		Staffing shortages	Q29: “What they explained to me was, they had so many people for one nurse, and if they only got four nurses up there, they only have so many people until somebody gets discharged.”
		Difficulty obtaining appointments	Q30: “I haven’t had a primary doctor since then until recently. It is hard to find one.”
		Long wait times	Q31: “Yeah, that’s a problem with her (doctor), and it’s frustrating when you get somewhere, and you’ve got an appointment. You’re supposed to be in there at 8:00, 8:30 and then just 9:00, 10 o’clock before you get to see the doctor.”
		Health team communication breakdown	Q32: “The first time they put me in (hospital) and did that, even the doctor that came in the next night, she got mad because she thought nobody told (doctor) I was here. She is in the same practice with the guy that put me in. He didn’t even let her (doctor) know I was there.”
			Q33: “Today they messed up. Yes, they had me getting up early, going to the (building), and they said I didn’t have an appointment there. So yes, they messed up today.”
		Impacting workers in the system	Q34: “She's (social worker) been trying to help us; she’s got the run around, too. It’s a mess in the system.”
Lack of insurance coverage consequences	60%	Discrimination based on insurance status	Q35: “About three, four months going back and forth to the hospital. If you don’t have insurance… They don't care about you if you haven't got insurance.”
		Inadequate care	Q36: “They actually asked if we had insurance at first anyways, but we tell them no. They kept sending him home. We just kept going back, calling the ambulance; going back.”
		Delays or denial of care	Q37: “That’s why it happened. I don’t have no insurance, so I kept putting it off. You can’t see a doctor without insurance.”
			Q38: “What makes it really hard is if you don’t have insurance. If you don’t have insurance, they kind of give you the runaround. Don't do the stuff that really needs to be done to catch the cancer for you.”
		Limited access to treatment	Q39: “I do not. I just haven’t had insurance. I don’t have insurance, so I never go to the doctor.”
*Policy*			
Health care access policies	68%	Barriers related to disability access	Q40: “What’s frustrating is 90% of the people I’ve talked to said that I should qualify for disability because I'm stage four cancer. But I know nothing about that and Change Healthcare for USA has been helping and trying to get that done. It’s still been going on and on.”
		Lack of access to universal health care	Q41: “[We should] have free health care. I mean too bad it’s not like England. You know how (in) England everybody gets free health care.”
Insurance policies	50%	Insurance limitations	Q42: “I don’t normally get sick, so I didn’t keep a doctor. Before my wife got a job with a second insurance, we were living off of my disability, and I couldn’t afford, but about one copay a month. So, I had to be really choosy with who I went to see.”
		High cost of insurance	Q43: “It’s because my employment doesn’t offer it (insurance). I've looked at (other insurance), and it’s just been too expensive… Most of the reatment that I have is usually out of pocket.”

PCP, primary care providers; Q, quote

^a^Percentage shows percentage of patients who reflected each theme.

Personal factors also played a role, particularly among male participants, who reported reluctance in seeking medical care, with one participant stating: “Men are stubborn about going to the doctor, and I kept refusing and refusing” (Q3).

### Category 2: Interpersonal-Level Barriers

Interpersonal barriers that emerged from the participant interviews included communication challenges, mistrust for health care providers, and absence of social support. Communication challenges were reported by 47% of the participants and included inadequate communication due to use of complex medical terminology: “When he tells me what’s going on, … I don't understand it in doctor’s terms” (Q16). Other reported that interpersonal barriers included inadequate information transfer between providers, lack of timely and transparent communication, lack of continuity between providers, and miscommunication regarding diagnosis: “… you just lose track of your doctors in the process” (Q17).

Mistrust toward physicians was another significant theme, reported by 20% of the participants. Some patients felt that their concerns were dismissed or that they were given conflicting information about their diagnosis and treatment. Subthemes included past negative experiences, perceived inconsistencies in care, and a lack of connection with their providers: “I don’t have doctors really truly because I don’t even know their names. … They are not there long enough to really get to know them” (Q22). Absence of social support also was felt by 27% of the patients, who expressed feelings of isolation and inadequate support: “I’ve got my phone, … but let's face it, my phone doesn’t ring that much. I don’t have a lot of people that I talk to” (Q23).

### Category 3: Organizational-Level Barriers

Organizational barriers reported included health system mistrust, inadequate health care infrastructure, and lack of insurance coverage consequences. Lack of insurance or under-insurance consequences were reported by 60% of the participants. Patients cited inadequate care, discrimination based on insurance status, delays in care, and limited access to treatment as consequences: “That’s why it happened. I don’t have no insurance so I kept putting it off. You can’t see a doctor without insurance” (Q37). One participant described being passed around: “… if you don’t have insurance, they kind of give you the runaround, don’t do the stuff that really needs to be done to catch the cancer for you” (Q38). This highlights the systemic hurdles faced by patients when seeking care without adequate insurance coverage.

Furthermore, both health system mistrust and inadequate infrastructure were reported by 37% of the patients. Inadequate health care infrastructure was reported to have multiple downstream consequences, including difficulties finding a PCP, staffing shortages, difficulty obtaining appointments, long wait times, delays in care, and health system communication breakdown.

### Theme 4: Policy-Level Barriers

Barriers in health care access policies, reported by 68% of the patients, were predominantly related to structural challenges in accessing health care, insurance limitations, and navigating disability support systems: “… I should qualify for disability because I'm stage four cancer… It’s still been going on and on” (Q40). One participant noted: “[We should] have free health care” (Q41) after discussing the financial burden imposed on him in the absence of comprehensive health care. Participants with advanced care reported challenges securing disability benefits. Insurance policies, including limitations of insurance and high costs, were cited by 50% of the participants as the main barriers: “… I've looked at it, and it’s just been too expensive” (Q43).

## Discussion

This mixed-methods study identified key barriers in access to cancer care among patients with foregut cancer treated at a safety-net hospital and tertiary care academic center in southern Alabama. Barriers were identified at the individual, interpersonal, organizational, and policy levels. By integrating qualitative insights with quantitative data, this study highlighted the complex interplay of barriers contributing to cancer care disparities in this population, particularly in a highly disadvantaged region such as the Deep South.

At the individual level, transportation challenges emerged as the most frequently reported barrier. Other individual level barriers reported were lack of access to PCP, financial barriers, caregiving and competing responsibilities, absence of social support, and personal barriers.

These qualitative findings align with our quantitative data, which show that patients traveled long distances to access their cancer care and may explain the delays in access to care that these patients experienced. The logistical complexities of traveling long distances to access specialized cancer care, particularly for patients living in a rural state such as Alabama,^21^ are compounded by patients’ lack of proximity to the health care system, resulting in significant delays to treatment initiation, which can have a significant impact on treatment options and outcomes.

Two participants reported insurance-related delays in care. However, the majority of the participants did not discuss the long times to access care despite the significant delays they noted based on electronic health record data. This discrepancy suggests that patients may have limited understanding or expectations regarding care. Less than half of the patients without a PCP in our study identified this as an issue. Research has shown that coordination of cancer care is highly influenced by the relationship between patients and their PCPs.^22^ Findings show that PCPs can help coordinate comprehensive cancer care, educate patients, and treat other common comorbidities among cancer patients, such as depression.^23^ Thus, increasing access to PCPs and strengthening patient engagement with primary care may enhance care coordination and improve outcomes. The majority of the patients who reported competing responsibilities did not receive GCT, suggesting that competing responsibilities are an important factor precluding patients from receiving optimal treatment. These patients should be identified early as having significant barriers to care and may require extra social and financial support.

Almost 50% of the participants reported challenges with physician communication related to the use of complex medical terminology and the reception of inconsistent information from different physicians. Participants reported feeling lost during transitions between providers and an absence of connection with a central, responsible physician. These challenges may collectively erode patient trust and contribute to confusion, possibly influencing adherence to treatment. Provider training in cultural responsiveness, patient-centered communication, using simplified language, and ensuring that information is conveyed in an accessible manner are potential strategies to improve physician–patient communication. Developing health literacy-sensitive educational materials and incorporating visual aids could be additional initiatives to improve physician–patient communication. Perceived or fundamental inconsistencies in communication highlight the need for better communication and coordination between health care teams. Ensuring that patients have access to a central point of contact, such as a patient navigator, can help address these issues.^24^

The organizational-level challenges highlighted were delays in accessing care and reception of inadequate care or no care due to lack of insurance. Although insurance access is a broader structural issue, access to care or the lack thereof at the health system level is a distinct challenge for patients who are uninsured or underinsured.^25^ Approximately one fourth of the participants in this study were uninsured, which is higher than the national average of 7.7%.^26^ These findings are consistent with patterns at other safety-net hospitals, which serve populations experiencing higher socioeconomic deprivation and limited access to health care resources. Safety-net hospitals also frequently have limited infrastructure and face additional strains, including resource allocation challenges, staffing shortages, and fewer support services, that exacerbate existing barriers for the population of patients most at risk of suboptimal or inadequate care.^27^ Together, these findings indicate that patients who are uninsured or underinsured face multiple barriers resulting in suboptimal cancer care pathways.

Medical mistrust may be influenced by historical injustices, previous negative interactions, patients’ limited access to health care, and disparities in health. Studies have shown this medical mistrust to be higher among black patients.^28–30^ Other studies reporting perspectives of black patients also have reported similarly high levels of distrust of the health system.^29,31^ At the health system level, acknowledging and addressing historical and systemic factors that contribute to mistrust, developing and strengthening community engagement, and incorporating patient-centered, culturally sensitive communication training are avenues to address medical mistrust.

This study expanded on the literature by providing a nuanced, multi-level analysis of barriers to foregut cancer care, particularly within the context of a safety-net hospital serving an underserved population in the Deep South.^32^ The cancer institute serves a high percentage of minority and underinsured patients, with 37% of the patients receiving cancer care at the cancer institute identified as black and 78% having insurance coverage other than private insurance.^10^ Almost one fourth of the study participants were uninsured. Thus, this study captures the perspectives of patients with significant barriers in access to care, and its findings have several important implications for physicians, health systems, and policymakers.

At the clinical level, improving patient navigation services and providing targeted support for transportation and financial challenges faced by patients may mitigate some individual-level barriers. Enhancing physician–patient communication and fostering trust through consistent, patient-centered care can address interpersonal challenges. Although several of the elucidated barriers may be addressed at the level of the health care team and hospital system, upstream public policy to address systemic barriers to cancer care is crucial. Findings have shown Medicaid expansion to be associated with improved access to care and better outcomes for patients with foregut cancers.^33–36^ Medicaid expansion has demonstrated the greatest positive impact on safety-net hospitals.^37–39^ and could provide the critical financial support necessary to expand patient-centered services. If adopted in Alabama, this could open a path for institutions to fund interventions aimed at improving access to GCT. It also would reduce the burden of uncompensated and under-compensated care faced by safety-net hospitals in the state.

In this study, several limitations were encountered. As a single-institution study in a southern state, its results may not be generalizable to other health care systems or geographic regions. Nevertheless, the identified barriers are likely to be relevant to disadvantaged patients with foregut cancer. Although this study used purposive sampling to ensure a diverse range of patient experiences, the perspectives captured were limited to patients willing and able to participate. The exclusion of non–English-speaking participants may have omitted important perspectives, particularly from immigrant populations who may face unique barriers to care. Although we recruited patients from a wide range of ages and diverse backgrounds, the relatively small sample precludes generalization of findings to patients without foregut cancer or patients underrepresented in the current study (i.e., black female participants) or those receiving care at smaller community centers).

Additionally, qualitative research is not intended to produce statistically generalizable results. The percentages reported in this study serve to provide additional descriptive detail about the study population and the relative prominence of different barriers among participants and cannot be extrapolated beyond this sample or be assumed to represent the prevalence of barriers in the broader population of patients with foregut cancers. Although this study provided rich qualitative insights from the patient perspective, it may have failed to capture the full scope of challenges from the perspective of caregivers or other health system stakeholders.

Finally, the cross-sectional nature of this study resulted in the capture of patient perspectives on barriers at a single time point and may have failed to adequately capture the longitudinal effects of some barriers. Future research should build on these findings and explore additional viewpoints to develop a more comprehensive understanding of barriers to foregut cancer care.

## Conclusion

This study highlights the multi-level barriers faced by patients with foregut cancer in accessing and adhering to GCT, particularly in underserved populations. Understanding patient perspectives is the critical first step to identifying barriers to care and developing strategies tailored to mitigate these barriers. By addressing challenges at the individual, interpersonal, organizational, and policy levels, physicians and health care systems can develop more effective, patient-centered interventions to reduce disparities and improve outcomes.

## Supplementary Information

Below is the link to the electronic supplementary material.

Supplementary file1 (PDF 573 kb)

Supplementary file2 (DOCX 24 kb)
