# Supplementary material for: Patient-Reported Barriers to Foregut Cancer Care in the Deep South
Source: Ann Surg Oncol. 2025 Mar 4;32(6):3900–12. doi: 10.1245/s10434-025-17113-2 (PMC12049283; doi:10.1245/s10434-025-17113-2)
Supplement: Supplementary file 2 — Supplementary file2 (DOCX 24 kb) [file 10434_2025_17113_MOESM2_ESM.docx]

**Barriers to Oncologic Care Interview Guide**

| **I’d like to talk about your experience with your cancer treatment.** |
| --- |
| 1. How has your experience with the medical system been, from the time you started having symptoms of cancer until now? |
| 1. How satisfied are you with the medical care you received after your cancer diagnosis?   IF NOT MENTIONED:  ◻ What makes you say satisfied/unsatisfied [depending on prior response]? |
| 1. About how long was it from the time your symptoms started to when you received your cancer diagnosis? 2. How satisfied are you with the care you received from the time your symptoms started to when you received your cancer diagnosis?   IF NOT MENTIONED:  ◻ What makes you say satisfied/unsatisfied [depending on prior response]? |
| 1. What makes it hard to follow up on your cancer treatment? (*Alternative wording:* What makes it hard for you to get treatment?)   IF PATIENT SEEMS UNSURE:  ◻ Other people have said that some things make it hard for them to get their cancer treatment. Sometimes people have trouble being able to get an appointment to see a cancer doctor, getting to doctor’s appointments, following doctors’ recommendations, or getting medications. Has anything like that been a problem for you?  ◻ You said __________ [list what they identified]. Can you tell me more about that?    ◻ How are you dealing with this?  ◻ Where are you getting help from? |
| **Next, I am going to ask about some of the things other people have said make it harder or easier to follow up with cancer treatment. (ONLY ASK ABOUT THINGS THAT THEY DIDN’T MENTION)** |
| 1. How helpful is your cancer care team when issues come up?   IF PATIENT SEEMS UNSURE: By cancer care team I mean all the people who help treat your cancer both medically and surgically  ◻ Can you tell me more about that? *If helpful:* What do they do that is helpful?    ◻ *If not helpful:* Can you tell me more about that?  ◻ *If not helpful:* How are you dealing with this? Where are you getting help from? |
| 1. Do you feel like your cancer doctors and cancer care team listens to you?   ◻ *If no:* Can you tell me more about that?    ◻ *If no:* How are you dealing with this? Where are you getting help from? |
| 1. Do you have problems with getting to and from your appointments? (Examples include: issues with travel distance)   ◻ *If yes:* Can you tell me more about that?    ◻ *If yes:* How are you dealing with this? Where are you getting help from?  *Follow up:* how long does it typically take you to get to your appointment? |
| 1. Do you have problems with treatment costs or payment?   (Examples include: co-pays, cost of transportation, loss of wages for you or for family members)  ◻ *If yes:* Can you tell me more about that?    ◻ *If yes:* How are you dealing with this? Where are you getting help from?  *Follow up:* Do you have insurance? If no: Has this been an issue? |
| 1. Do you have problems with how long your doctor’s appointments usually take?   ◻ *If yes:* Can you tell me more about that?    ◻ *If yes:* How are you dealing with this? Where are you getting help from?  FOR LONG APPOINTMENTS:  ◻ How do you plan for the timing for a doctor’s appointment?  ◻ How do you use that time at the doctor’s office?  IF NOT MENTIONED:  ◻ How do you feel before the appointment? How do you feel afterward? |
| 1. Do you have problems understanding your doctors’ instructions?   ◻ *If yes:* Can you tell me more about that?    ◻ *If yes:* How are you dealing with this? Where are you getting help from? |
| 1. Do you have problems remembering your doctors’ instructions?   ◻ *If yes:* Can you tell me more about that?    ◻ *If yes:* How are you dealing with this? Where are you getting help from? |
| 1. How much help do you have from family or friends?   ◻ Can you tell me more about that? *If helpful:* What do they do that is helpful?    ◻ *If not helpful:* Can you tell me more about that?  ◻ *If not helpful:* How are you dealing with this? Where are you getting help from? |
| 1. Do you have difficulties dealing with worry, anxiety, or sadness?   ◻ *If yes:* Can you tell me more about that?    ◻ *If yes:* How are you dealing with this? Where are you getting help from? |
| 1. Do you have problems with discrimination?   ◻ *If yes:* Can you tell me more about that?    ◻ *If yes:* How are you dealing with this? Where are you getting help from? |
| 1. Do you have problems with pain?   ◻ *If yes:* Can you tell me more about that?    ◻ *If yes:* How are you dealing with this? Where are you getting help from? |
| 1. Do you have problems getting and taking your medications?   ◻ *If yes:* Can you tell me more about that?    ◻ *If yes:* How are you dealing with this? Where are you getting help from? |
| 1. Do you have problems with treatment side effects?   IF SIDE EFFECTS ARE A PROBLEM:  ◻ Can you tell me more about that?    ◻ Have you called your doctor’s office to follow up on a side effect?  ◻ *If yes:* How did you feel before the phone call? How did you feel after you received a response?  ◻ *If no:* What made you not call the doctor’s office to follow up on a side effect?  FOR ALL RESPONDENTS:  ◻ Did your doctor talk to you about the side-effects that you should expect before starting treatment?  ◻ *If yes*: How was that discussion? Did you understand what was explained to you?  ◻ *If yes:* Would you change anything about that discussion you had with your doctor? |
| 1. In general, how do you get medical advice in between appointments if a problem comes up? (*Note: Assess ease of access, smoothness of process, etc.)*   ◻ What would you like to change about how you can get medical advice? |
| 1. How helpful is the hospital at addressing things that make it harder for you to get cancer treatment?   IF NOT MENTIONED:  ◻ How is the hospital helping?  ◻ How would you like them to help?  ◻ What could the hospital do to help you more? |
| **Next, I am going to ask you questions about your doctor(s) and care team.** |
| 1. How is your relationship with your cancer doctors and cancer care teams?   IF PATIENT SEEMS UNSURE: By cancer care team I mean all the people who help treat your cancer both medically and surgically  ◻ Can you tell me more about it? |
| 1. How much trust do you have for the medical system?   ◻ Can you tell me more about it? |
| 1. How much trust do you have for your cancer doctors?   ◻ Can you tell me more about it? |
| 1. How comfortable are you asking them questions and telling them your concerns?   ◻ Can you tell me more about it?  If no: what would make you more comfortable? |
| 1. Do you have a primary care physician?   ◻ *If yes:* What could your primary care team do to help you more?  ◻ *If no:* Would you like to establish care with a primary care physician? (IF YES: What help would you need to make that happen?) |
| **I’d like to talk about what helps with your cancer treatment.** |
| 1. In general, what currently helps your cancer treatment? This could be anything   ◻ Can you tell me more about that? |
| **Next, I am going to ask about some of the things other people have said help them through their cancer treatment. (ONLY ASK ABOUT THINGS THAT THEY DIDN’T ALREADY MENTION)** |
| 1. Do you have support from family or friends?   ◻ *If yes:* How helpful has this been for you?  ◻ *If no:* How helpful would this be for you? |
| 1. Do you attend support groups?   ◻ *If yes:* How helpful has this been for you?  ◻ *If no:* How helpful would this be for you? |
| 1. Have you talked to other cancer survivors?   ◻ *If yes:* How helpful has this been for you?  ◻ *If no:* How helpful would this be for you? |
| 1. Have you talked with a counselor?   ◻ *If yes:* How helpful has this been for you?  ◻ *If no:* How helpful would this be for you? |
| 1. Do you receive help from a social worker?   ◻ *If yes:* How helpful has this been for you?  ◻ *If no:* How helpful would this be for you? |
| 1. Do you receive help from a patient care advocate? (*if necessary, say*: When I saw patient care advocate, I mean a professional who could help coordinate your care, connect you with resources, and advocate for your healthcare needs?)   ◻ *If yes:* How helpful has this been for you?  ◻ *If no:* How helpful would this be for you? |
| 1. Do you have a relationship with your medical providers?   ◻ *If yes:* How helpful has this been for you?  ◻ *If no:* How helpful would this be for you? |
| 1. Do you receive help dealing with worry or sadness?   ◻ *If yes:* How helpful has this been for you?  ◻ *If no:* How helpful would this be for you? |
| 1. Have you talked through your cancer treatment goals with your cancer care team?   ◻ *If yes:* How helpful has this been for you?  ◻ *If no:* How helpful would this be for you? |
| 1. Have you been told what to expect from your cancer treatment?   ◻ *If yes:* How helpful has this been for you?  ◻ *If no:* How helpful would this be for you? |
| 1. Do you receive help with transportation?   ◻ *If yes:* How helpful has this been for you?  ◻ *If no:* How helpful would this be for you? |
| 1. How helpful is reading information provided to you by your doctor? For example, has your doctor given you any information to read?   ◻ *If yes:* How helpful has this been for you?  ◻ *If no:* How helpful would this be for you? |
| 1. Have you used any online resources or information?   ◻ *If yes:* How helpful has this been for you?  ◻ *If no:* How helpful would this be for you? |
| 1. Do you receive written, electronic or other instructions and reminders about tests and appointments?   ◻ *If yes:* How helpful has this been for you?  ◻ *If no:* How helpful would this be for you? |
| 1. Do you identify as spiritual or religious?   ◻ *If yes:* How helpful is your faith or beliefs in getting you through your cancer treatment?  ◻ *If no:* How helpful would this be for you? |
| 1. Have you talked to someone about end-of-life care?   ◻ *If yes:* How helpful has this been for you? How does talking about it make you feel?  ◻ *If no:* How helpful would this be for you? |
| 1. What other things do you think would help you as you go through your cancer treatment? |
| 1. What other things could be done to improve your overall cancer treatment? |
| 1. What would you change about the process of calling your doctor’s office? |
| 1. What advice would you give to someone else trying to receive cancer treatment? |
| 1. What else do you think would be helpful for us to know so that we can help others with their cancer treatment? |

**Thank you for your time and answers to these questions.**
